# DISC1 Regulates the Proliferation and Migration of Mouse Neural Stem/Progenitor Cells through Pax5, Sox2, Dll1 and Neurog2

**DOI:** 10.3389/fncel.2017.00261

**Published:** 2017-08-29

**Authors:** Qian Wu, Weiting Tang, Zhaohui Luo, Yi Li, Yi Shu, Zongwei Yue, Bo Xiao, Li Feng

**Affiliations:** ^1^Department of Neurology, First Affiliated Hospital, Kunming Medical University Kunming, China; ^2^Department of Neurology, Xiangya Hospital, Central South University Changsha, China; ^3^Department of Neurology, University of Massachusetts Medical School Worcester, MA, United States; ^4^Department of Neurology, The Second Xiangya Hospital, Central South University Changsha, China; ^5^Department of Neurology, Yale University School of Medicine New Haven, CT, United States

**Keywords:** DISC1, proliferation, migration, Pax5, Sox2, Dll1, Neurog2

## Abstract

**Background**: Disrupted-in-schizophrenia 1 (DISC1) regulates neurogenesis and is a genetic risk factor for major psychiatric disorders. However, how DISC1 dysfunction affects neurogenesis and cell cycle progression at the molecular level is still unknown. Here, we investigated the role of DISC1 in regulating proliferation, migration, cell cycle progression and apoptosis in mouse neural stem/progenitor cells (MNSPCs) *in vitro*.

**Methods**: MNSPCs were isolated and cultured from mouse fetal hippocampi. Retroviral vectors or siRNAs were used to manipulate *DISC1* expression in MNSPCs. Proliferation, migration, cell cycle progression and apoptosis of altered MNSPCs were analyzed in cell proliferation assays (MTS), transwell system and flow cytometry. A neurogenesis specific polymerase chain reaction (PCR) array was used to identify genes downstream of *DISC1*, and functional analysis was performed through transfection of expression plasmids and siRNAs.

**Results**: Loss of DISC1 reduced proliferation and migration of MNSPCs, while an increase in DISC1 led to increased proliferation and migration. Meanwhile, an increase in the proportion of cells in G0/G1 phase was concomitant with reduced levels of DISC1, but significant changes were not observed in the number MNSPCs undergoing apoptosis. *Paired box gene 5* (Pax5), *sex determining region Y-box 2* (Sox2), *delta-like1* (Dll1) and *Neurogenin2* (Neurog2) emerged as candidate molecules downstream of DISC1, and rescue experiments demonstrated that increased or decreased expression of either molecule regulated proliferation and migration in DISC1-altered MNSPCs.

**Conclusion**: These results suggest that Pax5, Sox2, Dll1 and Neurog2 mediate DISC1 activity in MNSPC proliferation and migration.

## Introduction

**Figure 1:**
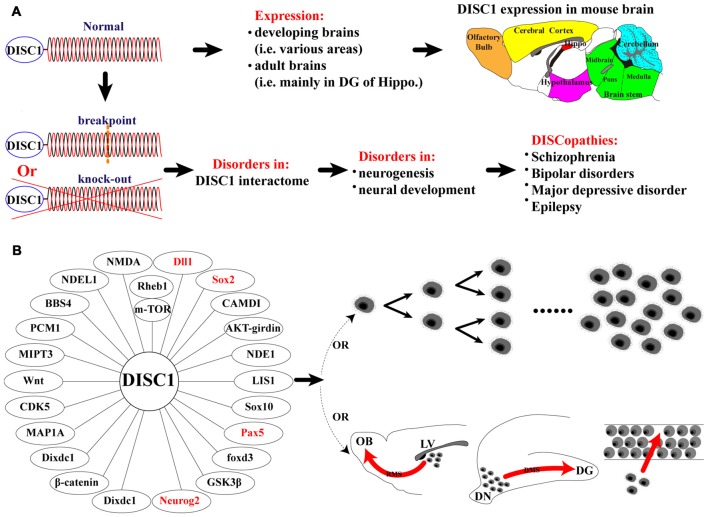
Summary of findings of the present study and functions of disrupted-in-schizophrenia 1 (DISC1) on proliferation and migration. **(A)** Schematic representation of normal DISC1 protein and the translocation in the affected Scottish family, and physiological and pathological conditions for normal and disrupted DISC1; **(B)** Schematic representation of proteins that are known to interact with DISC1 regulating the proliferation and migration of mouse neural stem/progenitor cells (MNSPCs), neurons and other cell types.

Until recently, many psychiatric disorders were undefined at the molecular level. These included schizophrenia (SZ; Le Strat et al., 2009), bipolar disorder (Blackwood et al., 2001b), major depressive disorder (Brandon et al., 2009) and epilepsy (Fournier et al., 2009, 2010). The discovery of *disrupted-in-schizophrenia 1* (*DISC1*) as a genetic risk factor for these disorders marked a breakthrough in understanding psychiatric illness. Genetic analysis of a Scottish family revealed that family members affected by SZ inherited a balanced translocation between chromosomes 1 and 11 (t(1:11) (q42.1:q14.3); Millar et al., 2000), which encodes a protein now known as DISC1 (Blackwood et al., 2001a; Millar et al., 2004). DISC1 dysfunction is confirmed as a mechanism underlying these psychiatric disorders, now broadly termed “DISCopathies” (Figure 1A; Korth, 2009).

DISC1 regulates almost all aspects of neurogenesis and neural development at various stages of brain development (Kang et al., 2011; Wu et al., 2013; Yerabham et al., 2013; Muraki and Tanigaki, 2015; Lee et al., 2015), including proliferation, migration, differentiation, neurite growth, morphogenesis and synaptogenesis. The molecular interaction network involving DISC1 has been referred to as the “DISC1 interactome” and plays a major role in neurogenesis and neural development (Figure 1A; Camargo et al., 2007; Korth, 2009). Members of this network include DISC1 binding partners such as Nuclear distribution protein nudE-like 1 (NDEL1), Fasciculation and elongation protein zeta-1 (FEZ1), lissencephaly (LIS1), Glycogen synthase kinase 3 beta (GSK3β), Girdin, Dixdc1 (Dixin), Growth factor receptor-bound protein 2 (Grb2), Ras homolog enriched in the brain 1 (Rheb1) and intracellular/extracellular signaling pathways, such as the AKT-mTOR, ERK, GSK3β, Wnt, NMDA-R, Reelin and GABA pathways (Fournier et al., 2010; Kang et al., 2011, 2015; Kim et al., 2012; Wu et al., 2013).

Despite our understanding of the DISC1 interactome (Camargo et al., 2007), few studies have focused on the role of DISC1 in the proliferation, migration, cell cycle progression and apoptosis of mouse neural stem/progenitor cells (MNSPCs). In this investigation, embryonic mouse hippocampal NSPCs were used as an *in vitro* cellular model to overexpress or silence *DISC1* with retroviral expression vectors or siRNAs, and the biology of these altered MNSPCs was examined in a series of functional assays. To further understand the mechanisms of DISC1 regulated neurogenesis, a neurogenesis quantitative polymerase chain reaction (PCR) array was used to identify potential genes or signaling pathways associated with altered expression of *DISC1*. *Paired box gene 5* (*Pax5*), *sex determining region Y-box 2* (*Sox2*), *Delta-like1* (*Dll1*) and *Neurogenin2* (*Neurog2*) were among the candidate genes identified and were further examined in functional assays to determine whether they mediated *DISC1* activity.

## Materials and Methods

### Ethics Statement

Animal protocols were approved by the ethics committee associated with Central South University (Changsha, Hunan, China). All experiments were performed in accordance with official recommendations by the Chinese animal community, and efforts were made to minimize the number of animals used and animal suffering.

### Mice

C57/BL6 pregnant female mice (*n* = 4; P 14.5; Slac Laboratory Animal Co. Ltd, Shanghai, China) were used for the isolation and culture of MNSPCs.

### Cell Culture

MNSPCs were isolated from the hippocampus of C57/BL6 fetal mouse brains as detailed in previous experiments (Yoneyama et al., 2011; Louis et al., 2013). Pregnant female mice (P 14.5) were anesthetized by i.p. injection with 10% chloral hydrate (0.1–0.2 mL/10 g), then the fetal mice were isolated from the uterus. The hippocampi of fetal mice were isolated using a dissecting microscope (Olympus). The hippocampi were washed in sterile cold phosphate buffered saline (PBS) in a 10 cm petri dish and cut into pieces using tissue scissors. The tissues were blown gently into smaller pieces using a 1 mL pipette, transferred into a 15 mL centrifuge tube, then D-HBSS was added, mixed into 10 mL suspension, and the tissues were incubated in room temperature (RT; 20–25°C) for 20 min with 1 mL ACCUTASE (A6964, Sigma). The tissue chunks were then spun down in a low-speed centrifuge at 700 *g* for 1 min at RT, and the supernatant was removed and transferred to a new 15 mL centrifuge tube. We determined the number of cells using a blood cell counting plate, seeded the cells at a density of 1 × 10^5^/mL in a petri dish, and cultured the cells in NeuroCult™ Prol iferation Kit (Stem Cell; Vancouver, BC, Canada) to keep them in an undifferentiated, proliferative state.

### siRNA Interference

Five siRNAs for each gene were designed against the functional sequences of *DISC1*, *Pax5, Sox2, Dll1 and Neurog2* and sense and antisense sequences were chemically synthesized (Sigma). MNSPCs were seeded at a density of 2 × 10^5^/mL in 24-well plates, and after 24 h were transfected with siRNAs at a concentration of 100 nM in Lipofectamine 2000 Reagent (Invitrogen, New York, NY, USA). The medium was replaced 48 h later, and the efficiency of gene knockdown was evaluated using real-time PCR (RT-PCR; Supplementary Figure S1).

### Retroviral Infection

#### Packaging of Retroviral Vectors and Determination of Titers

A full-length mouse *DISC1* sequence was ligated into the retroviral vector pCAG-EGFP (a generous gift from Dr. Chunmei Zhao, Laboratory of Genetics, The Salk Institute for Biological Studies, La Jolla, CA, USA; also available for purchase from Addgene; Cambridge, MA, USA), linearized with *Pmel* and *Sfil* restriction sites to generate the DISC1 expression construct CAG-DISC1-EGFP. The DISC1 retroviral construct and pCAG-EGFP were packaged into 293T cells with packaging plasmids pCMV-vsv-g and pCMV-gp (Addgene) and titered (as high as 10^8^ c.f.u./mL) as previously described (Tashiro et al., 2006; Zhao et al., 2006; Jessberger et al., 2007).

#### Multiplicity of Infection (MOI)

Viral titers were serially diluted (10^4^, 10^5^ and 10^6^) and applied to MNSPCs to determine the multiplicity of infection (MOI) prior to experimentation. MOI was assessed under fluorescence microscopy with expression of EGFP encoded by the retroviral vector. More than 60% of the MNSPCs were EGFP-positive after incubation with virus for 4 days at 10^4^ c.f.u./mL and an MOI of 100.

#### Infection of Cells

MNSPCs (2 × 10^5^/mL) were plated, and the medium was replaced 24 h later with 0.5 mL of 10^4^ c.f.u./mL diluted, filtered virus containing polybrene at a concentration of 6 μg/mL (Sigma; St. Louis, MO, USA). The medium was changed after 2 days, and *EGFP*-positive colonies were collected for experiments.

### Transfection of Cells by Electroporation

Expression plasmids for *Pax5* (pPax5), *Sox2* (pSox2), *Dll1* (pDll1) and *Neurog2* (pNeurog2) were constructed. One hundred microliter of cell suspension (10^7^ cells/mL) was pipetted into a 4-mm electroporation cuvette and plasmids were added to a final concentration of 100–200 nM (unless otherwise specified) immediately before electroporation. Electroporation was performed with a Fischer electroporator (Fischer; Heidelberg, Germany) with a rectangle pulse of 330 V for 10 min. Cells were incubated for 15 min at RT, diluted 20-fold with culture medium, and incubated at 37°C in 5% CO_2_.

### Real-Time PCR (RT-PCR)

RT-PCR was used to assess interference efficiency 48 h after cells were transfected with retrovirus vector, plasmids, or siRNAs. Total RNA was isolated with Trizol Reagent per the manufacturer’s protocols (Life Technologies; Grand Island, NY, USA). The samples were incubated with 1 μL DNase I (Life Technologies) for 30 min at 37°C to remove contaminating genomic DNA. cDNA was synthesized from RNA (200 ng) in a 20 μL reverse transcription reaction (Promega; Madison, WI, USA). RT-PCR was performed with SYBR Green qPCRSuperMix (Life Technologies) with the recommended program of one cycle at 95°C for 15 min, followed by 40 cycles at 95°C for 30 s and 60°C for 30 s on the ABI PRISM^®^ 7500 Sequence Detection System. Primers used for RT-PCR of *DISC1, Pax5, Sox2, Dll1* and *Neurog2* are listed in Table 1.

**Table 1:** Pairs of primer sequences for Disrupted-in-schizophrenia 1 (DISCI1), Paired box gene 5 (Pax5), sex determining region Y-box 2 (Sox2), deltalike1 (Dll1) and Neurogenin2 (Neurog2).

Name	Fwd	Rev
DISC1	5′CTCGGAGCCATGTACAGTCA 3′	5′ACCAGCTGTCGGATAGGAA 3′
Pax5	5′CAACAGGATCATTCGGACAA 3′	5′AGGATGCCACTGATGGAGTA 3′
Sox2	5′CAAGATGCACAACTCGGAGAT 3′	5′TCATGAGCGTCTTGGTTTTC 3′
Dll1	5′CTGTGGACTATAACCTCGTT 3′	5′ATCTTACACCTCAGTCGCTA 3′
Neurog2	5′ATGGTCAAAGAGGACTATGG 3′	5′ATTCCCTCTGAGAGATTCAC 3′
GAPDH	5′GGCCTCCAAGGAGTAAGAAA 3′	5′GCCCCTCCTGTTATTATGG 3′

Relative expression levels were calculated as ratios normalized with glyceraldehyde-3-phosphate dehydrogenase (*GAPDH*) expression. The RT-PCR data were analyzed using the ∆Ct method (Wang et al., 2010), and results were determined as the mean ± SD of three independent experiments.

### Western Blotting

Equal protein quantities of lysates prepared from MNSPCs were subjected to SDS-PAGE and transferred to PVDF membranes. Membranes were blocked for 2 h in blocking buffer (5% non-fat dry milk in PBS with 0.1% Tween 20) at RT, incubated overnight at 4°C with primary antibodies (rabbit anti-DISC1 (1:1000; Life Technologies), rabbit anti-Pax5 (1:1000, Abcam; Cambridge, MA, USA), rabbit anti-Sox2 (1:1000, Cell Signaling Technology; Danvers, MA, USA), rabbit anti-Dll1 (1:500, Abcam), and rabbit anti-Neurog2 (1:10,000, Abcam)), followed by incubation with a conjugated secondary antibody (goat anti-rabbit IgG-HRP (1:10,000; Thermo Scientific; Pittsburgh, PA, USA)). Membranes were rinsed three times for 10 min after each incubation with Tris-buffered saline (TBS) containing 0.1% Tween-20. Protein bands were visualized using ECL reagent (Thermo Scientific), and densitometric analysis of protein bands was performed with Image-Pro Plus 6.0 software. All western blotting data are representative of at least three independent experiments.

### Cell Proliferation Assay (MTS Assay)

MTS assay was performed to detect proliferation of MNSPCs. Approximately 10,000 cells in 100 μL of culture medium were seeded into three 96-well plates. Proliferation was determined at 0 h, 24 h, 48 h and 72 h following a 4 h incubation with 10 μL of MTS (3-(4,5-dimethylthiazol- 2-yl)-5-(3-carboxymethoxyphenyl)-2-(4-sulfophenyl)-2H-tetra- zolium) solution (2 mg/mL, Promega, G3582). Colorimetric evaluation was performed at 490 nm in a mELISA microplate reader (Thermo Fisher Scientific, Waltham, MA, USA).

### Flow Cytometric Detection of Apoptosis

Trypsinized cells (without EDTA) were rinsed twice in PBS and re-suspended in 1x binding buffer at a concentration of 1 × 10^6^ cells/mL. Cells (100 μL of solution containing ~1 × 10^5^ cells) were transferred to a 5 mL culture tube, and Annexin V-FITC antibody (5 μL) and propidium iodide (5 μL; BD Biosciences; San Jose, CA, USA) were added and incubated for 15 min at RT in the dark. Binding buffer (1×; 400 μL) was added, and the samples were analyzed by flow cytometry within 1 h on the FACSCalibur (Becton Dicknson; Franklin Lakes, NJ, USA).

### Cell Cycle Analysis

Cells were harvested, rinsed twice with PBS, and re-suspended in the residual PBS. Fixation was performed with cold 75% ethanol (10 mL) added dropwise while vortexing, and cells were incubated at −20°C for a minimum of 2 h. Cells were pelleted by centrifugation, rinsed twice with PBS, and adjusted to a final concentration of 1 × 10^7^ cells/mL. Aliquots (100 μL; 1 × 10^6^ cells) of the cell suspension were then transferred into 12 × 75 mm tubes and incubated with PI-PBS (500 μL; 50 μg/mL PI, 100 μg/mL RNase A, 0.2% Triton X-100) after gentle vortexing for 30 min at 4°C. Samples were analyzed by flow cytometry on the FACSCalibur (Becton Dicknson, Franklin Lakes, NJ, USA), and data were analyzed with ModFit LT software (Becton Dickinson, Franklin Lakes, NJ, USA).

### Transwell Migration Assay

Trypsinized cells suspended in serum-free DMEM medium were seeded onto untreated BD Falcon Cell Culture Inserts (8.0 μm Polyethylene terephthalate (PET) membranes; 0.8 × 10^6^ pores 1 cm^2^) at a density of approximately 2.5 × 10^4^ cells/cm^2^. The inserts were placed in the wells of BD Falcon multi-well plates containing 600 μL 10% FBS DMEM and incubated at 37°C in 5% CO_2_. After 24 h and 48 h, the cells were wiped off of the inserts with a cotton swab. The remaining cells migrating to the other side of the membrane were fixed with 4% paraformaldehyde (PFA) for 15 min and stained with Crystal Violet for 10 min, and then the average number of those cells was calculated to evaluate their migrated ability. Images were captured with the Olympus microscope.

### Mouse Neurogenesis PCR Array

The Mouse Neurogenesis RT^2^Profiler™ PCR Array (PAHM 404Z, Qiagen, Shanghai, China) was used to profile 84 genes related to the process of neurogenesis (Supplementary Table S1). RNA was isolated with the RNasy Mini Kit and RNase-Free DNase Set (Qiagen; Valencia, CA, USA). The RT^2^ First Strand Kit (Qiagen) was used to synthesize cDNA, and RT-PCR was performed using SYBR Green Master mixes with the arrays on the Applied Bio-systems 7500HT system per the manufacturer’s instructions. Expression of each gene was normalized against the following endogenous control genes: β-actin (*Actb*), β2 microglobulin (*B2m*), *GAPDH*, β-Glucuronidase (*Gusb*), and heat shock protein 90 α, cytosolic, class B (*Hsp90ab1*). The Ct values of all the wells were exported to a blank Excel^®^ spread sheet and analyzed with the SABiosciences (Qiagen) PCR Array Data Analysis Web-based software (RT^2^ Profiler PCR Array Data Analysis version 3.5).

### Statistical Analysis

All statistical analyses were performed with Statistical Package for the Social Sciences (SPSS Inc. 2007, version 16.0; Chicago, IL, USA). Results are expressed as the mean ± SD. Data were analyzed with Student’s *t*-test, and *p*-value < 0.05 were considered statistically significant.

## Results

### Silence or Overexpression of DISC1 and Pax5, Sox2, Dll1 and Neurog2

To investigate the effect of *DISC1*, *Pax5*, *Sox2*, *Dll1* and *Neurog2* on various biological parameters *in vitro*, siRNAs were transfected into MNSPCs to knock down expression of the corresponding genes. Using RT-PCR, decreases in endogenous mRNA levels of *DISC1*, *Pax5, Sox2*, *Dll1* and *Neurog2* were observed in MNSPCs. *DISC1*siRNA (100 nM), *Pax5*siRNA (50 nM) and *Sox2*siRNA (100 nM), *Dll1*siRNA (50 nM), and* Neurog2*siRNA (50 nM) were effective at inhibiting the expression of *DISC1*, *Pax5*, *Sox2*, *Dll1* and *Neurog2*. mRNA levels decreased by 90%, 72.5%, 72.7%, 80.7% and 80.9% respectively (Supplementary Figure S1).

**Figure 2:**
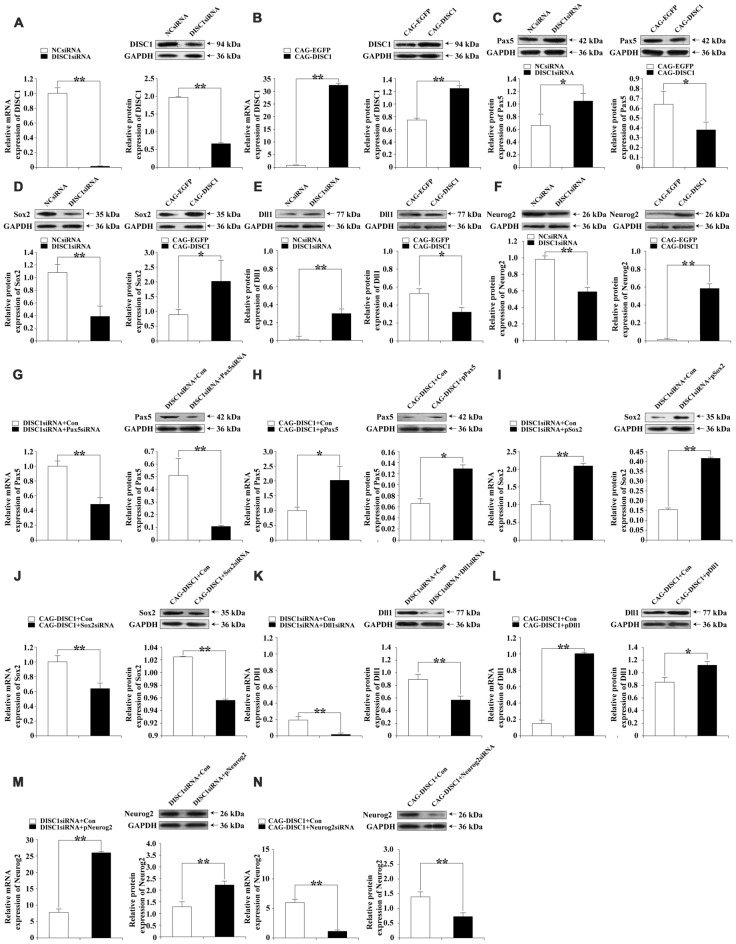
mRNA and protein expression levels of DISC1, Paired box gene 5 (Pax5), sex determining region Y-box 2 (Sox2), delta-like1 (Dll1) and Neurogenin2 (Neurog2) in knockdown and exogenous expression experiments. Real-time polymerase chain reaction (RT-PCR) and Western blotting were performed on cells where gene expression was modulated with siRNA, plasmid, or retroviral vector. RT-PCR and Western blots are paired according to the molecule analyzed for each gene interference experiment, except in **(C–F)** where only Western blots are shown. Bar graphs are used to display quantification by RT-PCR and Western blotting. The relative intensities of DISC1, Pax5, Sox2, Dll1 and Neurog2 by Western blot were normalized to the internal reference protein glyceraldehyde-3-phosphate dehydrogenase (GAPDH; KangChen Bio-tech, Shanghai, China). **(A)** DISC1, DISC1siRNA; **(B)** DISC1, CAG-DISC1; **(C)** Pax5, DISC1siRNA (Left); Pax5, CAG-DISC1 (Right); **(D)** Sox2, DISC1siRNA (Left); Sox2, CAG-DISC1 (Right); **(E)** Dll1, DISC1siRNA (Left); Dll1, CAG-DISC1 (Right); **(F)** Neurog2, DISC1siRNA (Left); Neurog2, CAG-DISC1 (Right); **(G)** Pax5*, DISC1*siRNA + *Pax5*siRNA; **(H)** Pax5, CAG-*DISC1* + p*Pax5*; **(I)** Sox2, *DISC1*siRNA + p*Sox2*; **(J)** Sox2, CAG-*DISC1* + *Sox2*siRNA; **(K)** Dll1*, DISC1*siRNA + *Dll1*siRNA; **(L)** Dll1, CAG-*DISC1* + p*Dll1*; **(M)** Neurog2, *DISC1*siRNA + p*Neurog2*; **(N)** Neurog2, CAG-*DISC1* + *Neurog2*siRNA *p < 0.05; **p < 0.01 (controls-NCsiRNA, CAG-EGFP, DISC1siRNA + Con, CAG-DISC1 + Con). All results are from three independent experiments.

To determine whether the siRNAs (negative control siRNA (NCsiRNA) and DISC1siRNA) and expression constructs (CAG-EGFP and CAG-DISC1) affected protein levels, DISC1 protein was examined by Western blotting. RT-PCR and Western blotting were performed to detect the expression of DISC1 48 h after transfection with siRNAs or the expression constructs. siRNA led to decreases in DISC1 mRNA (Figure 2A, Left) and protein (Figure 2A, Right) levels by 53.2-fold and 2.9-fold, respectively, compared to controls. In contrast, transfection of the retroviral vector CAG-DISC1 increased mRNA (Figure 2B, Left) and protein (Figure 2B, Right) expression by 32.5- and 1.7-fold respectively. Changes in protein levels demonstrated that protein function may be altered as a result of transfection with siRNA or the expression construct.

### DISC1 Promotes Proliferation of MNSPCs

**Figure 3:**
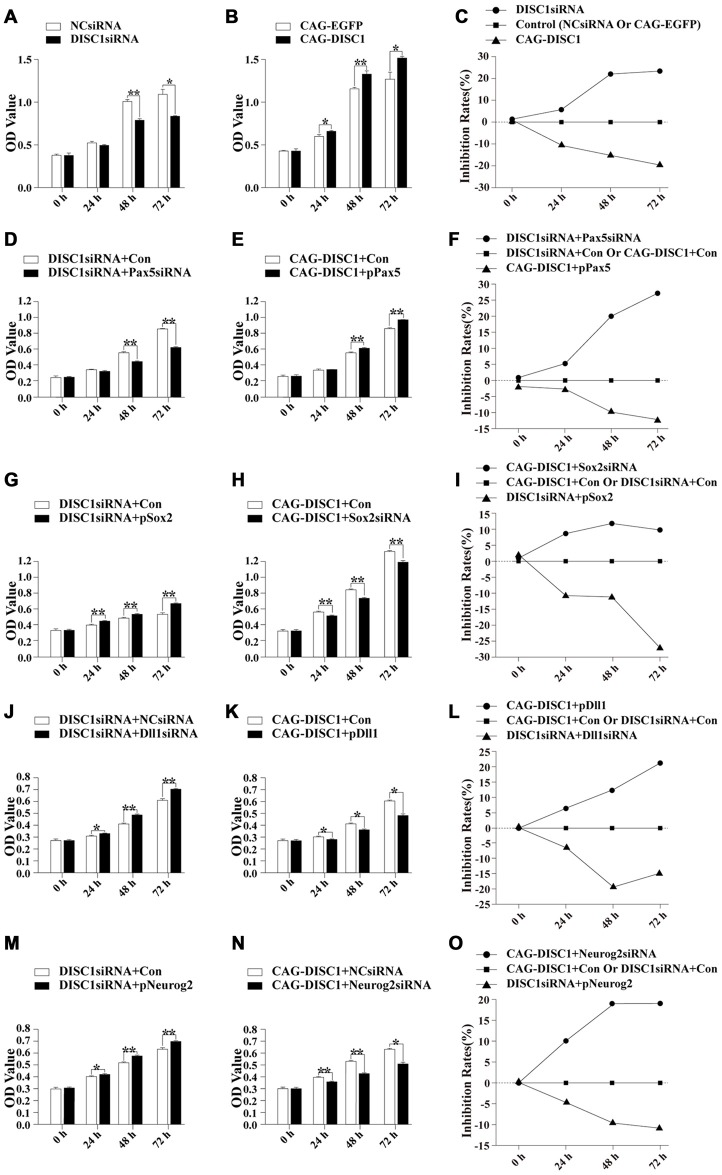
DISC1 promotes proliferation of MNSPCs. Bar graphs representing OD values derived from MTS assays at 0, 24, 48, and 72 h and line charts are used to present rates of inhibition in different gene interference experiments in MNSPCs. Interference is performed with siRNA and/or expression vectors indicated. **(A)** OD, *DISC1*siRNA; **(B)** OD, CAG-*DISC1*; **(C)** inhibition rates by line chart, *DISC1*siRNA and CAG-*DISC1*; **(D)** OD, *DISC1siRNA* +* Pax5*siRNA; **(E)** OD, CAG-*DISC1* + p*Pax5*; **(F)** inhibition rates presented in line charts, *Pax5*siRNA or p*Pax5*; **(G)** OD, *DISC1*siRNA + p*Sox2*; **(H)** OD, CAG-*DISC1* +* Sox2*siRNA; **(I)** inhibition rates presented in line charts, *Sox2*siRNA or p*Sox2*; **(J)** OD, *DISC1*siRNA + *Dll1*siRNA; **(K)** OD, CAG-*DISC1* + p*Dll1*; **(L)** inhibition rates presented in line charts, *Dll1*siRNA or p*Dll1*; **(M)** OD, *DISC1*siRNA + p*Neurog2*; **(N)** OD, CAG-*DISC1* +* Neurog2*siRNA; **(O)** inhibition rates presented in line charts, *Neurog2*siRNA or p*Neurog2*. *p < 0.05, **p < 0.01. The rate of proliferation is expressed as a relative percentage of inhibition, which was calculated as follows: cell growth/proliferation inhibition rates (%) = [(A control − A sample)/A control] × 100% (controls-NCsiRNA, CAG-EGFP, DISC1siRNA + Con, CAG-DISC1 + Con). All results are from three independent experiments.

To examine the role of DISC1 on MNSPC proliferation, MTS assays were performed in cells transfected with siRNAs or expression construct. Proliferation of MNSPCs significantly diminished after siRNA knock-down of *DISC1*, whereas proliferation increased with increased *DISC1* expression. We observed that siRNA inhibited proliferation of MNSPCs over time by 5.7%, 22% and 23.4% after 24 h, 48 h and 72 h respectively. In contrast, increased expression of *DISC1* promoted cell growth by 10.6%, 15.2% and 19.6% after 24 h, 48 h, and 72 h, respectively (Figures 3A–C). These results indicate that the *DISC1* gene had a role in promoting growth of MNSPCs in a time-dependent manner.

### DISC1 Promotes MNSPC Migration

**Figure 4:**
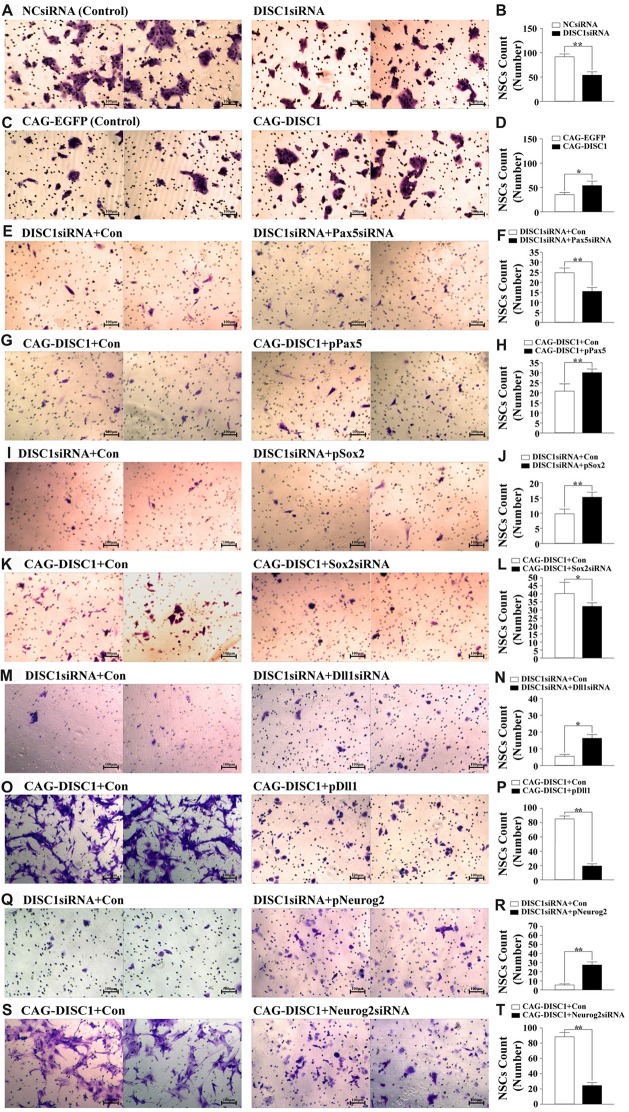
DISC1 promotes the migration of MNSPCs. Migration of control or altered MNSPCs was examined in transwell assays, and migrated cells were stained with crystal violet. Migrated cell counts are represented as bar graphs. Gene interference molecule (siRNA or expression construct) are as indicated. **(A)** Crystal violet stain, negative control siRNA (NCsiRNA) (control); *DISC1*siRNA; **(B)** bar graph, NCsiRNA (control) vs. *DISC1*siRNA; **(C)** crystal violet, CAG-EGFP (control); CAG-DISC1; **(D)** bar graph, CAG-EGFP (control) vs. CAG-DISC1; **(E)** crystal violet, *DISC1*siRNA (control); *DISC1*siRNA + *Pax5*siRNA; **(F)** bar graph, *DISC1*siRNA (control) vs. *DISC1*siRNA + *Pax5*siRNA; **(G)** crystal violet, CAG-DISC1 (control); CAG-DISC1 + p*Pax5*; **(H)** bar graph, CAG-DISC1 (control) vs. CAG-DISC1 + p*Pax5*; **(I)** crystal violet, *DISC1*siRNA (control)*; DISC1*siRNA + p*Sox2*; **(J)** bar graph, *DISC1*siRNA (control) vs.* DISC1*siRNA + p*Sox2*; **(K)** crystal violet, CAG-DISC1 (control); CAG-DISC1 + *Sox2*siRNA; **(L)** bar graph, CAG-DISC1 (control) vs. CAG-DISC1 + *Sox2*siRNA; **(M)** crystal violet, DISC1siRNA (control); DISC1siRNA + *Dll1*siRNA; **(N)** bar graph, DISC1siRNA (control) vs. DISC1siRNA +* Dll1*siRNA; **(O)** crystal violet, CAG-DISC1 (control); CAG-DISC1 + p*Dll1*; **(P)** bar graph, CAG-DISC1 (control) vs. CAG-DISC1 + p*Dll1*; **(Q)** crystal violet, DISC1siRNA (control); DISC1siRNA + p*Neurog2*; **(R)** bar graph, DISC1siRNA (control) vs. DISC1siRNA + p*Neurog2*; **(S)** crystal violet, CAG-DISC1 (control); CAG-DISC1 + *Neurog2siRNA*; **(T)** bar graph, CAG-DISC1 (control) vs. CAG-DISC1 +* Neurog2siRNA*. *p < 0.05, **p < 0.01 (controls-NCsiRNA, CAG-EGFP, DISC1siRNA + Con, CAG-DISC1 + Con). All results are from three independent experiments.

To determine how DISC1 influences migration, the ability of transfected MNSPCs to cross the Transwell membranes was examined. The average number of MNSPCs that migrated across the membrane after siRNA administration was fewer relative to controls (53.5 vs. 90.7 cells; *p* < 0.05; Figures 4A,B). *DISC1* overexpression, however, led to increased MSPNC migration relative to the control group (53.7 vs. 34.8 cells; *p* < 0.01; Figures 4C,D). These results show that *DISC1* mediates MNSPC.

### DISC1 Influences Apoptosis and Cell Cycle Progression of MNSPCs

**Figure 5:**
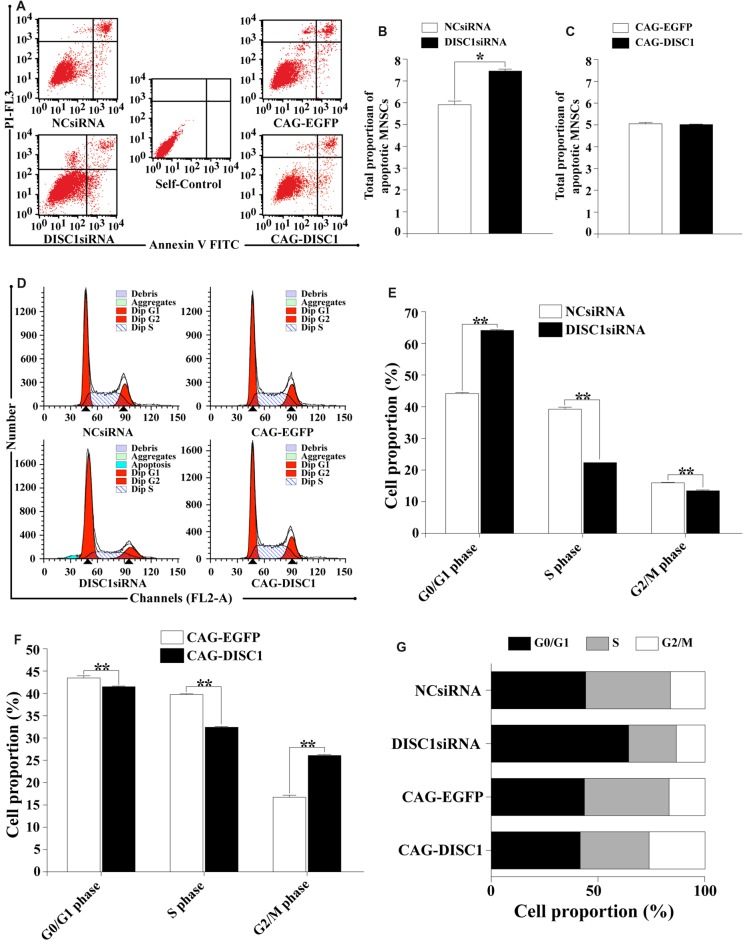
Interference of DISC1 expression alters apoptosis and cell cycle progression of MNSPCs. MNSPCs were analyzed by flow cytometry for apoptosis or cell cycle progression at the time points indicated following introduction of siRNA or expression constructs. **(A)** Measurement of apoptotic MNSPCs by flow cytometry after introduction of *DISC1*siRNA and CAG-DISC1. Quadrants for flow cytometry analysis are defined as the lower left (LL), lower right (LR), upper left (UL), and upper right (UR). Cells in LL (annexin V^−^-PI^+^) represent living cells; LR (annexin V^+^-PI^−^), early apoptotic cells or cytoclasis; UR (annexin V^+^-PI^+^), late apoptotic cells; UL (annexin V^−^-PI^−^), debris/cells with membrane only. UL + UR quadrants represent total MNSPCs undergoing apoptosis; **(B,C)** total proportion of MNSPCs undergoing apoptosis represented in bar graphs. *p < 0.05; **(D)** cell cycle analysis of MNSPCs transfected by NCsiRNA (control), DISC1siRNA, CAG-EGFP (control) and CAG-DISC1, respectively. Typical cell cycle profiles are presented where the first red peak corresponds to G1 phase, and the second peak, the G2 phase. The hatched blue region represents MNSPCs in S phase; **(E,F)** proportion of MNSPCs in G1/G0, S and G2/M presented in bar graphs. Constructs used are indicated; controls are compared to **(E)** *DISC1*siRNA and **(F)** CAG-DISC1. **p < 0.01; **(G)** cell cycle distribution of MNSPCs with *DISC1siRNA* or CAG-DISC1. Percentage of cells in G1 (black), S (gray), and G2/M (white) are represented in each bar for MNSPCs treated with the constructs indicated (controls-NCsiRNA, CAG-EGFP). All results are from three independent experiments.

Apoptosis is an important cell function inhibiting cell growth. Flow cytometry was used to determine how *DISC1* expression influences apoptosis in MNSPCs. Based on the total number of apoptotic events calculated in the four MNSPC groups, only *DISC1*siRNA altered apoptosis in MNSPCs. The percentage of MNSPCs undergoing apoptosis was greater with transfection of *DISC1*siRNA (7.5%) than NCsiRNA, CAG-EGFP and CAG-*DISC1* (5%–6%; Figures 5A–C). These results suggest that loss of *DISC1* only slightly increases apoptosis of MNSPCs.

Interestingly, both decreased and increased *DISC1* levels altered cell cycle progression. Decreased expression of *DISC1* inhibited cell growth as the average percentage of cells in G0/G1 phase increased from 44.2% to 64.2% (*p* < 0.01) and those in the S phase decreased from 39.7% to 22.3% at 48 h (*p* < 0.01). Cells in the G2/M phase had a decrease from 16.2% to 13.5% (*p* < 0.01; Figures 5D,E,G). In contrast, increased expression of *DISC1* reduced the average percentage of cells in the G0/G1 phase (43.5% to 41.5%; *p* < 0.01) as well as in the S phase (39.6% to 32.3%; *p* < 0.01) relative to controls. The proportion of cells in the G2/M fraction, however, increased from 16.9% to 26.2% (*p* < 0.01; Figures 5D,F,G). These results show that alterations in *DISC1* levels affect nearly all phases of the cell cycle in MNSPCs, with *DISC1* reduction resulting in some form of G1 arrest.

### DISC1 Levels Affect Neurogenic Specific Gene Expression

**Figure 6:**
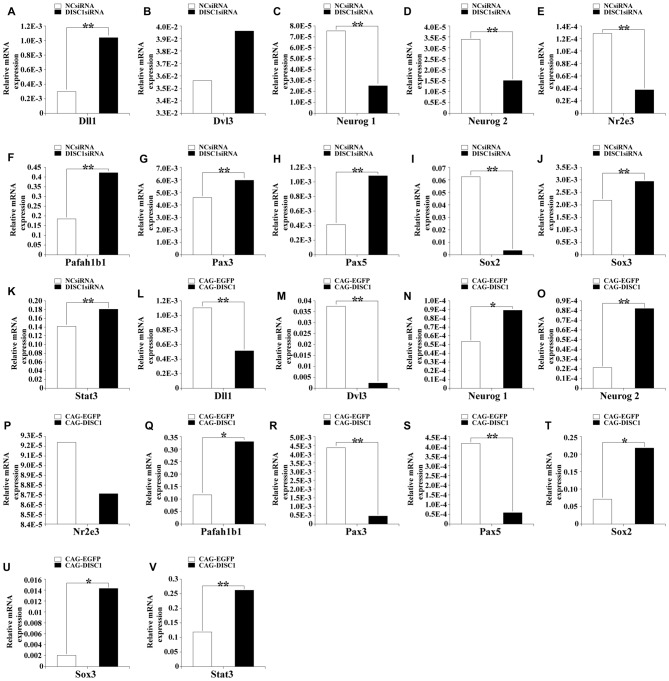
Pax5, Sox2, Dll1 and Neurog2 mRNA levels are regulated by DISC1, the levels were assessed by PCR array. Relative mRNA expression of several neurogenesis related genes after introduction of *DISC1*siRNA **(A–K)** or CAG-DISC1 **(L–V)** into MNSPCs (controls-NCsiRNA, CAG-EGFP). All results are from three independent experiments. *p < 0.05, **p < 0.01.

To identify candidate genes downstream of *DISC1*, expression levels of 84 neurogenesis-related genes in RNA isolated from transfected MNSPCs were determined using RT-PCR. In cells transfected with *DISC1*siRNA relative to controls, *Neurog1*, *Neurog2*, *Nr2e3*, and *Sox2* were significantly decreased (3.0-, 2.3-, 3.3- and 16.6-fold, respectively; *p* < 0.01), whereas *Dll1*, *Pafah1b1*, and *Pax5* were increased (3.4-, 2.3-, and 2.6-fold, respectively; *p* < 0.01; Figure 6). When *DISC1* was overexpressed, mRNA levels of *Dll1*, *Dvl3*, *Pax3*, and *Pax5* were down-regulated (2.1-, 14.3-, 9.1- and 6.9-fold, respectively; *p* < 0.01), whereas *Neurog2*, *Pafah1b1*, *Sox2*, *Sox3*, and *Stat3* mRNA levels were upregulated (3.8-, 2.8-, 3.0-, 6.6- and 2.2-fold, respectively; Figure 6; *p* < 0.01). Taken together, these results show that *Dll1*, *Neurog2*, *Pax5*, and *Sox2* are affected in opposite directions in response to increased or decreased DISC1 levels, while *Pafah1b1* expression increases under both experimental conditions (Figures 6A–V).

### Pax5 or Sox2 or Dll1 or Neurog2 Protein Levels Are Regulated by DISC1

Based on the results of neurogenesis PCR array, expression of *Pax5*, *Sox2*, *Dll1* and *Neurog2* was further examined at the protein level. Western blotting was performed to determine how the protein levels of Pax5, Sox2, Dll1, or Neurog2 are affected by expression changes in *DISC1* in MNSPCs. Transfection of MNSPCs with *DISC1*siRNA increased Pax5 (1.6-fold; *p* < 0.05; Figure 2C, Left) and Dll1 (9.7-fold; *p* < 0.01; Figure 2E, Left) protein levels after 48 h but decreased Sox2 (2.8-fold; *p* < 0.01; Figure 2D, Left) and Neurog2 (1.3-fold; *p* < 0.01; Figure 2F, Left) levels. In contrast, *DISC1* overexpression resulted in a decrease in Pax5 (1.7-fold; *p* < 0.05; Figure 2C, Right) and Dll1 (1.6-fold; *p* < 0.05; Figure 2E, Right) levels after 48 h, but an increase in Sox2 (2.2-fold; *p* < 0.05; Figure 2D, Right) and Neurog2 (29.5-fold; *p* < 0.01; Figure 2F, Right) levels. Overall, Pax5, Sox2, Dll1, and Neurog2 levels parallel mRNA expression, suggesting that DISC1 regulates the expression of Pax5, Sox2, Dll1 and Neurog2 at both mRNA and protein levels.

### Pax5, Sox2, Dll1 and Neurog2 siRNAs and Plasmids Alter mRNA and Protein Levels

RT-PCR and Western blotting were performed to determine mRNA and protein expression levels of Pax5, Sox2, Dll1, and Neurog2 48 h in MNSPCs where DISC1 levels were altered, after transfection with siRNA and expression plasmids. After transfection with their corresponding siRNA, *Pax5* mRNA and protein levels decreased (2.0-fold and 4.1-fold, respectively; Figure 2G), as did *Sox2* (1.6- and 1.1-fold, respectively; Figure 2J), *Dll1* (15.8- and 1.6-fold, respectively; Figure 2K), and *Neurog2* (5.9- and 1.9-fold, respectively; Figure 2N). Meanwhile, *pPax5* expression plasmid increased *Pax5* mRNA and protein levels (2.0- and 1.8-fold, respectively; Figure 2H), as did *Sox2* (2.1- and 2.6-fold, respectively; Figure 2I), *Dll1* (7.1- and 1.3-fold, respectively; Figure 2L), and *Neurog2* (3.3- and 1.8-fold, respectively; Figure 2M). These results show that siRNAs down-regulate the expression of *Pax5*, *Sox2*, *Dll1* and *Neurog2*, whereas *pPax5*, *pSox2*, *pDll1* and *pNeurog2* up-regulate the expression of *Pax5*, *Sox2*, *Dll1* and *Neurog2* in MNSPCs with increased or decreased *DISC1*.

### Proliferation of MNSPCs in Pax5, Sox2, Dll1 or Neurog2 Rescue Experiment

The previous results show that *DISC1*siRNA: (1) decreases MNSPC proliferation; and (2) increases Pax5 and Dll1 while decreasing Sox2 and Neurog2 expression. Based on these observations, rescue experiments were designed to examine whether DISC1 regulates the MNSPC proliferation via Pax5, Sox2, Dll1, Neurog2, or a combination of the candidates. We expected that in *DISC1*siRNA MNSPCs, down-regulation of *Pax5* by siRNA would enhance proliferation; surprisingly, proliferation was inhibited by 5.3%, 20.0%, and 27.1% at 24 h, 48 h and 72 h respectively. Conversely, we found that in CAG-*DISC1* MNSPCs, up-regulation of Pax5 increased proliferation by 2.7%, 9.8%, and 12.3% after 24 h, 48 h and 72 h. These results suggest that Pax5 regulates the proliferation of MNSPCs independent of DISC1 (Figures 3D–F).

In the *DISC1*siRNA MNSPCs, up-regulation of Sox2 increased proliferation by 10.8%, 11.3%, and 26.8% after 24 h, 48 h and 72 h (Figures 3G,I), down-regulation of Dll1 increased proliferation by 6.5%, 19.5% and 14.8% (Figures 3J,L), up-regulation of Neurog2 increased proliferation by 5%, 9.6% and 11.1% (Figures 3M,O). In contrast, in CAG-*DISC1* MNSPCs, down-regulation of Sox2 by siRNA inhibited proliferation by 8.6%, 11.8%, and 9.8% after 24 h, 48 h, and 72 h (Figures 3H,I), up-regulation of Dll1 inhibited proliferation by 6.7%, 12.2%, and 21.3% after 24 h, 48 h, and 72 h (Figures 3K,L), and down-regulation of Neurog2 inhibited proliferation by 10.0%, 18.9%, and 19.1% after 24 h, 48 h, and 72 h (Figures 3N,O). These results suggest that DISC1 and Sox2, Dll1 and Neurog2 co-regulate the MNSPC proliferation.

### MNSPC Migration after Pax5, Sox2, Dll1 or Neurog2 Rescue Experiment

Rescue experiments were performed to investigate whether DISC1 regulates MNSPC migration via Pax5, Sox2, Dll1 or Neurog2. In *DISC1*siRNA MNSPCs, down-regulation of *Pax5* by siRNA decreased the average number of migratory MNSPCs compared to the control (15.5 vs. 24.7; *p* < 0.01), contrary to our initial expectations. In CAG-*DISC1* MNSPCs, up-regulation of Pax5 increased the average number of migratory MNSPCs compared to the control (30.2 vs. 21; *p* < 0.01). These results suggest that Pax5 regulates the migration of MNSPCs independent of DISC1 (Figures 4E–H).

In *DISC1*siRNA MNSPCs, up-regulation of Sox2 increased the average number of migratory MNSPCs (15.3 vs. 9.8; *p* < 0.01; Figures 4I,J), down-regulation of Dll1 increased the average number of migratory MNSPCs (15.8 vs. 4.8; *p* < 0.05; Figures 4M,N), and up-regulation of Neurog2 increased the average number of migratory MNSPCs compared to control (27.8 vs. 5.3; *p* < 0.01; Figures 4Q,R). In contrast, in CAG-*DISC1* MNSPCs, down-regulation of Sox2 reduced the number of migratory MNSPCs (32 vs. 40; *p* < 0.05; Figures 4K,L), up-regulation of Dll1 decreased the average number of migratory MNSPCs (19 vs. 85.8; *p* < 0.01; Figures 4O,P), and down-regulation of Neurog2 reduced the average number of migratory MNSPCs compared to control (25 vs. 88.5; *p* < 0.01; Figures 4S,T). These results suggest that DISC1 regulates the migration of MNSPCs with Sox2, Dll1 or Neurog2.

## Discussion

We used an *in vitro* model to characterize the function of DISC1 during neural development. We show that DISC1 promotes the proliferation and migration of MNSPCs, and loss of this protein profoundly alters cell cycle progression does not significantly alter apoptosis. Furthermore, experiments with knockdown or overexpression of Pax5, Sox2, Dll1 and Neurog2 suggest that DISC1 regulates the MNSPC proliferation and migration through one or more of these transcription factors.

Our observation that DISC1 positively regulates the proliferation and migration of MNSPCs corroborates previous studies performed *in vivo* on the embryonic and developing brain (Brandon and Sawa, 2011; Ishizuka et al., 2011). Neuronal proliferation and migration are essential to the process of neurogenesis and neural development (Tomita et al., 2011). DISC1 mediates the proliferation of neuronal progenitors in the developing cortex through the Wnt/β-catenin pathway (Mao et al., 2009), thus the phosphorylation of DISC1 may be a key molecular event from maintaining the proliferation of mitotic progenitor cells to activating the migration of post-mitotic neurons in the developing cortex (Ishizuka et al., 2011). However, the function of DISC1 on neuronal migration is complicated. *DISC1* deficiency generally results in migration defects. For example, *DISC1* knockdown results in migration delay in the developing neocortex (Duan et al., 2007; Kubo et al., 2010), but, DISC1 has opposite effects on neuronal migration in the developing vs. adult hippocampal neurons. While *DISC1* deficiency delays migration in the developing hippocampal neurons, loss of *DISC1* induces inappropriate migration in newly generated neurons in the adult hippocampus (Duan et al., 2007; Enomoto et al., 2009; Kim et al., 2009). DISC1 function also affects migration in a temporal manner. *DISC1* knockdown does not alter migration of pyramidal cells in the developing CA1 at postnatal day 2 (P2; Meyer and Morris, 2009), whereas migration appears to be especially sensitive to *DISC1* suppression from P2 to P3 (Meyer and Morris, 2009; Tomita et al., 2011). Moreover, DISC1 is known to regulate migration in a cell type specific manner. Loss of function of DISC1 hinders granule cell migration in the embryonic hippocampus, but fails to influence neuronal migration of pyramidal neurons in CA1 (Meyer and Morris, 2009), and DISC1 seems to have no impact on postnatal neuroblast migration within the rostral migratory stream (RMS; Wang et al., 2011). Further complicating the issue is an underlying difference in mouse strains due to a spontaneous 25-base pair (bp) deletion of *DISC1*. Swiss Webster, ICR, and 129S6/SvEv mice, for example, lose production of some specific isoforms of DISC1, which leads to a reduced or no apparent effect on neuronal migration, compared to C57BL/6 mice without the deletion (Ishizuka et al., 2006; Koike et al., 2006; Meyer and Morris, 2009). Taken together, the influence of DISC1 on neuronal migration seems to be affected by temporal and spatial differences, cell type specificity, and animal model. Although DISC1 is known to interact with several protein partners and extracellular/intracellular signaling pathways to co-regulate mainly neuronal proliferation and migration (Figure 1B; Kamiya et al., 2005; Duan et al., 2007; Namba et al., 2011), the exact molecular mechanisms remain to be elucidated.

In the present study, dysregulation of DISC1 strongly affects cell cycle progression of MNSPCs but only moderately affects apoptosis (Figure 5). Expression of mutated *DISC1* has been previously associated with abnormalities in the expression of cell cycle genes and oligodendroglial progenitor markers (Katsel et al., 2011). *DISC1* suppression reduces MNSPC proliferation and leads to premature exit from the cell cycle and differentiation (Mao et al., 2009). In addition, a partner of DISC1, the FEZ1, has been associated with the cell cycle and apoptosis in neuronal development (Assmann et al., 2006). A role for DISC1 in the cell cycle and apoptosis is observed in diseases of the central nervous system (CNS). For example, mitochondria involved in apoptosis are the predominant site of DISC1 expression, suggesting that the effect of DISC1 on apoptosis is related to neuronal disturbances in the pathogenesis of SZ (James et al., 2004). Furthermore, DISC1 is shown to be a target of miR-181b, a miRNA which may have a role in the pathogenesis of SZ (Carroll et al., 2013). Therefore, understanding the role of DISC1 in the cell cycle and apoptosis in different cell types requires further investigation.

Expression array analysis reveals *Sox2, Pax5, Dll1 and Neurog2* as potential downstream or partner mediators of *DISC1* (Figures 6A,D,H,I,L,O,S,T). Overexpression of the gene leads to increased *Sox2 and Neurog2* but decreased *Pax5* and *Dll1* levels while suppression of *DISC1* induces the opposite expression profile (Figures 2C–F). Therefore, we propose that DISC1 regulates proliferation and migration through one or more of these transcription factors. The results of *Sox2, Dll1* and* Neurog2* interference in functional assays parallel *DISC1* expression, hence *Sox2*, *Dll1* and *Neurog2* appear to be necessary for proliferation and migration induced by *DISC1* (Figures 3G–O, 4I–T). However, alterations in *Pax5* expression yielded unexpected results in the functional assays, namely that increased *Pax5* also promoted proliferation and migration rather than inhibiting these functions in response to *DISC1* activity (Figures 3D–F, 4E–H). These results suggest that: (1) DISC1 regulates the proliferation and migration of MNSPCs through Sox2, Dll1 and Neurog2 and that exogenous intervention of *Sox2, Dll1* and* Neurog2* may help maintain the ability of MNSPCs to proliferate and migrate; and (2) Pax5 regulates the proliferation and migration of MNSPCs independent of DISC1, and endogenous changes in *Pax5* levels may be a protective factor against *DISC1* dysfunction, and thus Pax5 may act as a partner or co-regulator of DISC1.

*Sox2* is a transcription factor that is expressed in MNSPCs and mature neurons in the mouse CNS and is necessary to maintain self-renewal or pluripotency of undifferentiated embryonic stem cells (Fong et al., 2008). Activated Sox2 promotes cell proliferation and differentiation in diverse cell types (Ferri et al., 2004; Que et al., 2009; Tompkins et al., 2011), and changes in levels of *Sox2* transcription leads to abnormalities in the growth and development of stem cells (Bani-Yaghoub et al., 2006; Suh et al., 2007). In this study, Sox2 was also found to promote proliferation (Figures 3G–I) and migration (Figures 4I–L) of MNSPCs. Taken together, the data suggest that *DISC1* regulates the proliferation and migration of MNSPCs directly or indirectly through *Sox2*.

Pax5 was originally identified as the B-cell-specific activator protein (BSAP; Adams et al., 1992), a transcription factor expressed at early but not late stages of B lymphocyte differentiation (Urbánek et al., 1994; Eberhard and Busslinger, 1999). Although expression of this gene was originally associated with proliferation of immune cell types, the phenotype of knock-out mice reveals a role for Pax5 in neural development. Transient expression of Pax5 occurs during embryogenesis in the mesencephalon and spinal cord in a spatial and temporal manner (Adams et al., 1992). Functionally, *Pax5* over-expression reduces migration and motility of invasive MDA-MB231 cells, whereas the depletion of endogenous *Pax5* enhances the migration of MCF-7 cells (Vidal et al., 2010). Moreover, *Pax5* modulates the transcription of genes involved in the migration and adhesion of B cells, and promotes the adhesion of intercellular junctions in tumor cells (Schebesta et al., 2007). Our study found that Pax5 regulates the proliferation (Figures 3D–F) and migration (Figures 4E–H) of MNSPCs with DISC1. These findings suggest that Pax5 may stimulate proliferation and migration in a variety of cell types.

Notch ligand Dll1 may also be associated with DISC1-regulated neurogenesis. In the developing brain, steady *Dll1* expression inhibits the proliferation of neural progenitors and accelerates neurogenesis (Shimojo et al., 2016), whereas oscillatory expression of *Dll1* during embryonic development of the mammalian telencephalon is critical in neural fate decision (Barton and Fendrik, 2013) and cell differentiation in neural progenitor cells (Formosa-Jordan et al., 2012, Barton and Fendrik, 2013) through Notch signaling. In this study, Dll1 was found to regulate proliferation (Figures 3J–L) and migration (Figures 4M–P) of MNSPCs. Taken together, the data suggest that *DISC1* regulates the proliferation and migration of MNSPCs directly or indirectly through Dll1.

*Neurog2* is a member of the neurogenin subfamily of basic helix-loop-helix (bHLH) transcription factor genes that plays an important role in neurogenesis (Heng et al., 2008).* Neurog2* is involved in the regulation of proliferation (Vied et al., 2014), migration, and apical progression (Kawaue et al., 2014) of the ventricular zone during neocortical development, as well as the control of successive steps of neurogenesis in the embryonic cerebral cortex (Azzarelli et al., 2014; Li et al., 2014). In this study, we found that *Neurog2* also regulates proliferation (Figures 3M–O) and migration (Figures 4Q–T) of MNSPCs with DISC1, but how they work together requires further investigation.

The results from the expression array further suggest *Pax3*, *Sox3, Dvl3* and *Neurog1* as potential partners/mediators of *DISC1* in regulating the proliferation or migration of MNSPCs. These observations provide new molecular clues for future studies of the “DISC1 interactome”. *Pax3* mediates the migration of myoblasts in the developing limb bud through a signaling pathway which includes the activation of the c-Met (MET or Methylnitronitrosoguanidine HOS Transforming gene) receptor (Bottaro et al., 1991; Galland et al., 1992; Bober et al., 1994; Goulding et al., 1994; Bladt et al., 1995; Epstein, 1996). In addition, Pax3 is required for fine-tuning migration behavior of cardiac neural crest cells, but the protein is not essential for regulating their migration (Epstein et al., 2000). Pax3 was also found to be necessary for migration rather than differentiation in limb muscle precursors of mice (Daston et al., 1996). Although the exact role of Pax3 in MNSPC migration is unclear, *Pax3* is considered as a candidate gene associated with *DISC1* function based on our study.

Sox3 belongs to the high mobility group (HMG) family of transcription factors and is expressed in neural progenitor cells throughout the developing CNS. The protein is essential for the proliferation of neuroepithelial precursors (Rizzoti et al., 2004) and existing progenitors (Rogers et al., 2009). Furthermore, *Xnr5*, an early zygotic gene regulated by β-catenin/VegT, is a direct target of *Sox3*, suggesting that *Sox3* mediates cell proliferation through Wnt signaling pathways (Zhang et al., 2003). Taken together, DISC1 appears to shape an intricate network associated with Sox2, Sox3 and Wnt signaling pathways which regulate cell proliferation.

*Dvl3* is a Wnt signaling related gene whose mRNA levels changed in response to DISC1 in our study. A previous study reports that disheveled (Dvl; three isoforms) not only promotes β-catenin signaling in the Wnt pathway by stimulating GSK-3β phosphorylation but also promotes AKT signaling pathways by facilitating AKT phosphorylation. *DISC1* participates in the WNT signaling pathway and in WNT-mediated cell proliferation, either in primary neural stem/progenitor *in vitro* or in embryonic brains (Mao et al., 2009). In addition to *Dvl3, Pafah1b1* (also named *lis1*) has been shown to participate in neurodevelopmental processes such as neuronal precursor proliferation and differentiation, neuronal migration and neurite outgrowth, possibly in together with DISC1 through direct or indirect interactions. However, the exact mechanism remains to be defined (Lipska et al., 2006; Bradshaw et al., 2008, 2011; Rastogi et al., 2009; Bradshaw and Porteous, 2012). Additionally, *Neurog1* is shown to influence the differentiation and migration of neural crest stem/progenitor cells and fate specification of embryonic stem cells (Velkey and O’Shea, 2013). Whether *Neurog1* regulates neurogenesis via DISC1 requires further investigation.

## Conclusion

Our findings reveal an important role for DISC1 in neurogenesis. First, DISC1 is required for the proliferation and migration of MNSPCs: *DISC1* deficiency results in proliferation and migration defects, while *DISC1* overexpression promotes both activities. Second, DISC1 regulates the proliferation and migration of MNSPCs *in vitro* with Pax5, Sox2, Dll1, and Neurog2, which may act as downstream mediators of DISC1 activity. Third, Pax3, Sox3, Dvl3, Neurog1 are potential partners of DISC1 in the regulation of neurogenesis.

DISC1 appears to be a crucial regulator not only of neural development but also of specific neurogenesis-related genes/molecules and signaling pathways. Further investigation of the molecular components of the DISC1 regulatory complex, as well as the nature of more detailed molecular interactions of DISC1 with Pax5, Sox2, Dll1 and Neurog2 in regulating migration, proliferation and the processes of neurogenesis such as differentiation is needed.

## Author Contributions

QW: conception and design, collection and assembly of data, data analysis and interpretation, manuscript writing, final approval of manuscript, provision of study material. WT: conception and design, collection and assembly of data, data analysis and interpretation, final approval of manuscript. ZL: conception and design, data analysis and interpretation, final approval of manuscript. YL, YS and ZY: conception and design, data analysis and interpretation. BX and LF: conception and design, financial support, final approval of manuscript.

## Conflict of Interest Statement

The authors declare that the research was conducted in the absence of any commercial or financial relationships that could be construed as a potential conflict of interest.

## Supplementary Material

FIGURE S1 mRNA levels of Disrupted-in-schizophrenia 1 (*DISC1*), Paired box gene 5 (*Pax5*), and sex determining region Y-box 2 (*Sox2*) knocked down with siRNA. **(A–E)** siRNAs designed against each gene were transfected into cells and assessed for efficiency of knockdown by Real-time-polymerase chain reaction (RT-PCR); **(F)** Sense and anti-sense for each gene. All results are from three independent experiments. **p* < 0.05, ***p* < 0.01.
