# Supplementary material for: DISC1 Regulates the Proliferation and Migration of Mouse Neural Stem/Progenitor Cells through Pax5, Sox2, Dll1 and Neurog2
Source: Front Cell Neurosci. 2017 Aug 29;11:261. doi: 10.3389/fncel.2017.00261 (PMC5581844; doi:10.3389/fncel.2017.00261)
Supplement: Supplementary file 1 [file Table_1.pdf]

Table S1. 84 Neurogenesis related genes covered on the 96-well plate of PCR Array

Neurogenesis	Genes
Neuronal Migration	Ascl1, Cdk5r1, Dcx, Drd2, Ndn, Neurog2, Nrcam, Ntn1, Pafah1b1, Robo1, Slit2
Cell Differentiation	
Neuronal Differentiation	Ascl1, Bdnf, Bmp2, Bmp4, Cdk5r1, Cdk5rap2, Hes1, Heyl, Mef2c, Neurod1,Neurog1, Neurog2, Nog, Nrcam, Olig2, Pafah1b1, Pax3, Pou4f1, Rtn4, Sox2
Neuronal Cell Fate	Ascl1, Ntf3, Olig2, Sox2
Other Regulators	Hdac4, Mdk, Nrg1 (Hgl), Odz1, Pax5, Pax6
Synaptic Functions	
Synaptic Plasticity	Adora1, Apoe, Bdnf, Drd2, Grin1, Nf1, S100b
Synaptic Transmission	Apoe, Chrm2, Creb1, Dlg4 (Psd95), Drd2, Fgf2 (Bfgf), Grin1, Pafah1b1, Sod1, Th
Synaptogenesis:	Ache, Nrcam, Pou4f1
Axonogenesis	Apbb1, App, Dcx, Drd2, Erbb2 (Her2), Mtap2, Notch1, Nrcam, Pard3, Pou4f1, S100a6, S100b
Growth Factors & Cytokines	
Growth Factors	Artn, Bdnf, Egf, Fgf2 (Bfgf), Gdnf, Gpi1, Mdk, Ndp, Nrg1 (Hgl), Ptn, S100a6, Vegfa
Cytokines	Bmp2, Bmp4, Bmp8b, Cxcl1, Gpi1, Il3, Mdk, Ptn, Tgfb1
Apoptosis	Adora1, Adora2a, Alk, Apoe, Bcl2, Ep300, Gdnf, Notch2, Ntn1, Pax3, Rtn4, S100b, Vegfa
Cell Adhesion	Dll1, Efnb1, Nrcam, Nrp1, Nrp2, Rac1, Robo1, Slit2, Tnr
Cell Cycle	Apbb1, Ep300, Hdac4, Mdk, Mll1, Ndn, Pard3, Ptn
Signal Transduction	
Notch Signaling	App, Ascl1, Dll1, Hes1, Hey1, Hey2, Heyl, Notch1, Notch2, Nrg1 (Hgl)
WNT Signaling	Dvl3, Ndp, Shh
TGFß Signaling	Bmp2, Bmp4, Bmp8b, Tgfb1
G-Protein Coupled Receptor Signaling	Adora1, Adora2a, Chrm2, Cxcl1, Drd2
Transcription Factors & Cofactors	Apbb1, Ascl1, Creb1, Ep300, Flna, Hes1, Hey1, Hey2, Heyl, Mef2c, Mll1, Ndn, Neurod1, Neurog1, Neurog2, Nr2e3, Pax3, Pax5, Pax6, Pou3f3, Pou4f1, Sox2, Sox3, Stat3
