# Supplementary figures and images for: DISC1 Regulates the Proliferation and Migration of Mouse Neural Stem/Progenitor Cells through Pax5, Sox2, Dll1 and Neurog2
Source: Front Cell Neurosci. 2017 Aug 29;11:261. doi: 10.3389/fncel.2017.00261 (PMC5581844; doi:10.3389/fncel.2017.00261)

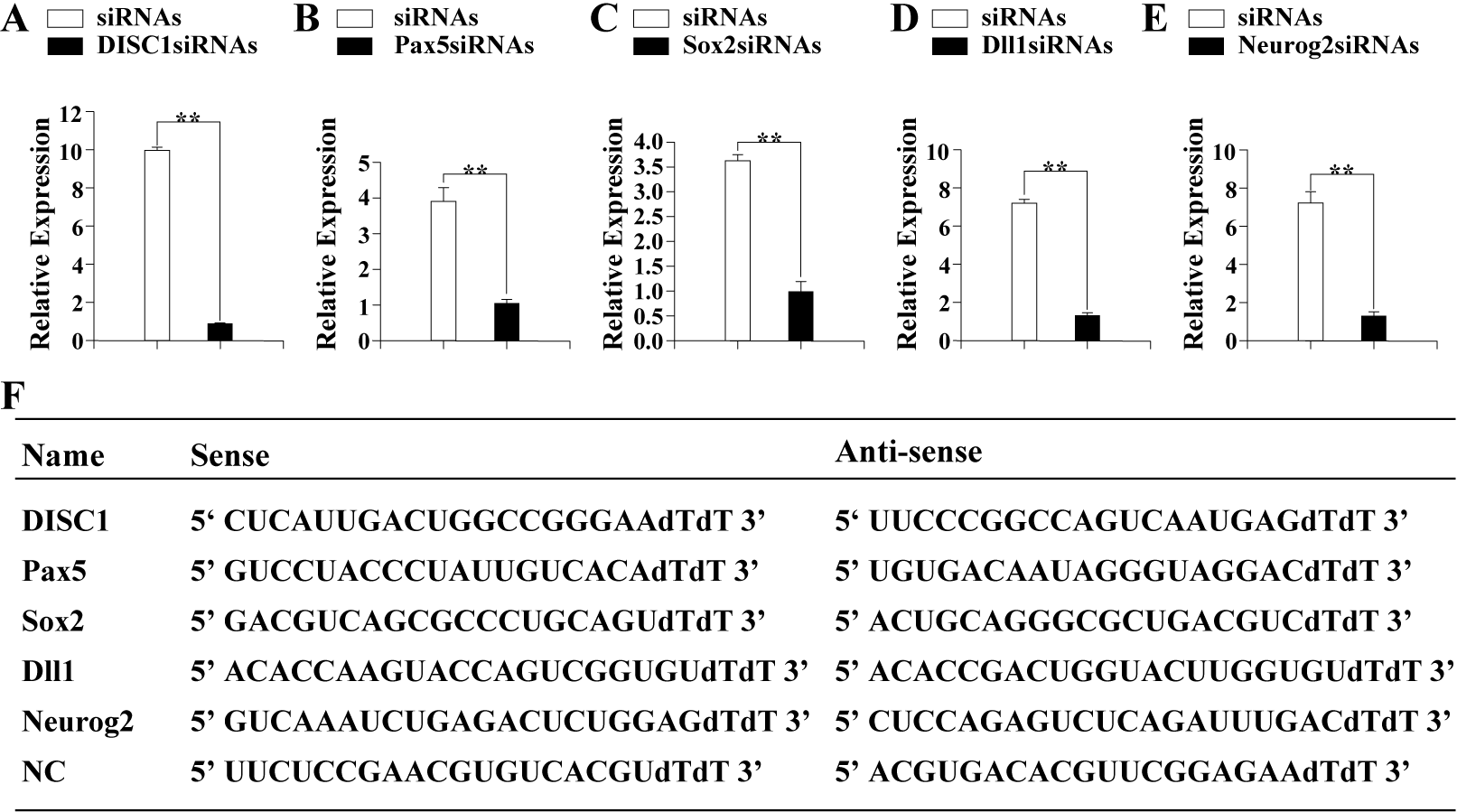

Supplement: FIGURE S1 — mRNA levels of Disrupted-in-schizophrenia 1 (DISC1), Paired box gene 5 (Pax5), and sex determining region Y-box 2 (Sox2) knocked down with siRNA. (A–E) siRNAs designed against each gene were transfected into cells and assessed for efficiency of knockdown by Real-time-polymerase chain reaction (RT-PCR); (F) Sense and anti-sense for each gene. All results are from three independent experiments. *p < 0.05, **p < 0.01. [file Image_1.tif]
